# Perceived barriers to early detection of breast cancer in Wakiso District, Uganda using a socioecological approach

**DOI:** 10.1186/s12992-018-0326-0

**Published:** 2018-01-23

**Authors:** Deborah Ilaboya, Linda Gibson, David Musoke

**Affiliations:** 1 Department of Social Work and Health, School of Social Sciences,, Nottingham Trent University, 50 Shakespeare Street, Nottingham, NG1 4FQ UK; 2 Department of Disease Control and Environmental Health, School of Public Health, College of Health Sciences, Makerere University, Kampala, Uganda

**Keywords:** Breast cancer, Barriers, Early detection, Socioecological framework, Uganda

## Abstract

**Background:**

Early detection of breast cancer is known to improve its prognosis. However, women in most low and middle income countries, including Uganda, do not detect it early hence present at an advanced stage. This study investigated the perceived barriers to early detection of breast cancer in Wakiso district, Uganda using a multilevel approach focused through a socioecological framework.

**Methods:**

Using qualitative methods, participants were purposively selected to take part in the study. 5 semi-structured interviews were conducted among the community members while two focus groups were conducted amongst women’s group and community health workers (CHWs) in Ssisa sub county, Wakiso district. In addition, 7 key informant interviews with health professionals, policy makers and public health researchers were carried out.

**Results:**

Findings from the study revealed that barriers to early detection of breast cancer are multifaceted and complex, cutting across individual, interpersonal, organizational, community and policy barriers. The major themes that emerged from the study included: knowledge, attitudes, beliefs and practices (KABP); health system and policy constraints; and structural barriers. Prominent barriers associated with KABP were low knowledge, apathy, fear and poor health seeking behaviours. Barriers within the health systems and policy arenas were mostly centred around competing health care burdens within the country, lack of a cancer policy and weak primary health care capacity in Wakiso district. Distance, poverty and limited access to media were identified as the most prominent structural barriers.

**Conclusion:**

Barriers to early detection of breast cancer are complex and go beyond individual behaviours. These barriers interact across multiple levels of influence such as organizational, community and policy. The findings of this study could provide opportunities for investment in multi-level interventions.

**Electronic supplementary material:**

The online version of this article (10.1186/s12992-018-0326-0) contains supplementary material, which is available to authorized users.

## Background

Breast cancer is an important public health challenge and is the leading cause of female cancer mortality globally, with an incidence of 25.1% [1]. Recent GLOBOCAN statistics revealed that breast cancer mortality rates in less developed regions of the world such as in sub-Saharan Africa (SSA) is approximately 62%, that is almost two-thirds of global mortality [2]. In Uganda, breast cancer is the second most commonly diagnosed female cancer after cervical cancer, with an estimated incidence rate of 15.8% and associated mortality of 11.4% of all female cancers [1]. These figures indicate high incidence and corresponding mortality due to the disease.

Women in Uganda and most low and middle income countries (LMICs) are disproportionately affected by high mortality associated with breast cancer, compared to their counterparts in western countries such as the United Kingdom, Canada or the United States of America [3]. Although the exact cause of this disparity is unknown [4], some studies have identified delayed detection which consequently results in advanced cases of breast cancer a major cause [5]. While early detection is known to improve breast cancer prognosis and offset costs associated with care [6], women in these LMICs present at an advanced stage, usually at stages III and IV characterized by large and almost incurable tumours, thereby reducing chances of survival [4]. Generally, Uganda has one of the highest rates of cancer globally [7], but there are limited studies on why these high rates occur. In Uganda, most cases of breast cancer are presented at an advanced stage. For example, a study of 244 patients revealed that peak presentation of breast cancer in Uganda was at stage III, 124 presented at stage III, 64 were metastatic at presentation while only 56 patients presented early [8]. However, the factors responsible for these delays have not been examined within the Ugandan context.

The concept of early detection of breast cancer is somewhat ambiguous [9]. According to the World Health Organization (WHO), the aim of early detection of breast cancer is to ensure that breast cancer is identified, referred and treated early [10], making it an important component of the breast cancer care pathway. Early detection measures recommended by the Breast Health Global Initiative guidelines (BHGI) for LMICs include: breast awareness and breast self-examination (BSE); clinical history and clinical breast examination (CBE); diagnostic breast imaging such as ultrasound; and mammography screening for high risk populations [11]. The most basic of these measures include BSE which simply involves getting to know one’s breast [12] and breast awareness which involves knowledge of the risk factors, signs and symptoms of breast cancer to prompt diagnosis and treatment [10]. Scientific evidence for the use of BSE as an individual early detection approach is controversial [13]. However, the simple clinical procedure of CBE is regarded as a preferred approach in comparison to BSE, mostly because it is carried out by trained health personnel and considered an effective method for early detection in LMICs [11]. A medical diagnostic method such as mammography is predominantly conducted among asymptomatic populations and is currently recommended for developed countries [14].

Despite the established significance of early detection of breast cancer, this disease is still not detected early in many SSA countries [4, 15] for several reasons. Previous studies have identified various barriers to early detection of breast cancer especially in SSA. For example, lack of knowledge, weak health systems, inadequate access to care and absence of functioning cancer registries are all identified as main barriers to early detection [4, 15]. The situation in these SSA countries is worsened as most of them grapple with a double burden of communicable and non-communicable diseases [15]. Although approaches to cancer detection generally in less developed countries tend to be individualistic and highly medicalised [16], these barriers cannot be adequately explained at the individual level. Hence, a socioecological framework was adopted to provide a comprehensive lens for examination.

The socioecological framework, a model put forward by McLeroy [17], has evolved as a health promotion model used for comprehensive health behaviour interventions [18]. The framework suggests that individual behaviours both shape and are shaped by wider structural factors, indicating synergistic interactions across five multiple levels of influence- individual, interpersonal, organizational, community and policy levels [18]. These levels also provide guidance on areas of focus for comprehensive health promotion interventions [17, 18]. This framework has been used for cancer screening studies [19] and colorectal cancer screening [20]. For this study, the framework provided a theoretical lens through which barriers to early detection of breast cancer can be examined within a wider realm of individual, interpersonal, organizational, community and policy level interactions. Therefore, recognizing that barriers to early detection of breast cancer in Uganda are not limited to individual behaviours, the objective of this study was to critically examine the perceived individual, interpersonal, organizational, community and policy barriers to early detection of breast cancer in a selected area of Uganda through a socio-ecological lens.

## Methods

### Study design

This study was a qualitative survey conducted through semi-structured interviews, focus group discussions (FGDs) and key informant interviews. A qualitative approach was deemed appropriate for this study because of its suitability in capturing the world of lived experience [21]. Thus, it provided well grounded, detailed description and explanation of the realities of the barriers to early detection of breast cancer within the Ugandan context. Question guides were designed in English and were used as prompts to facilitate interactions with participants. These question guides were designed based on the levels of the socioecological framework to allow participants to share perceived barriers within the multiple level of influences. The semi-structured interviews were designed as open-ended and flexible short conversations, and lasted approximately 30 min. The key informant interviews were more in depth and lasted for about 1 h. The focus groups lasted approximately 90 min and used semi-structured guides to allow for flexibility given the sensitivity of the topic under study. The question guides used prompts and comprised of four thematic sections: breast cancer awareness, early detection, barriers, and suggestions to improve early detection.

### Study setting

The study was conducted in Ssisa sub-county, Wakiso district in the central region of Uganda. Wakiso district encircles the capital city, Kampala. This district is the second largest in Uganda with an approximate population of 2,008,000 as indicated from the 2014 census [22]. Access to the study setting was provided through a project that was strengthening the capacity of community health workers in this area [23].

### Participants selection

The study participants were selected using purposive sampling based on two main criteria- being a woman and experience in healthcare delivery or health research particularly in relation to cancer care. The first criterion was used to select individuals for the semi-structured interviews and focus group for the women’s group, as the study was focused on female breast cancer. Participants for the semi-structured interviews were different from those for the focus group. Women who had been diagnosed with breast cancer or known to have a history of breast cancer were excluded from the study for ethical reasons. Secondly, experience was a key criterion during the selection of the key informants and community health workers (CHWs) focus group. CHWs were particularly selected for their experience in providing front line health care and delivering health promotion messages to community members. The semi-structured interviews and focus group participants were recruited through the help of a local community mobiliser who had worked in the sub-county for several years. Key informants were identified through established contacts both in the UK and Uganda.

### Data collection

Data was collected over a 3-week period in April 2015. Prior to commencement of data collection, a pilot study comprising of 7 female post graduate African students in the UK was conducted. This was to ensure the appropriateness of the data collection tools. Participants for the pilot study were selected through convenience sampling.

For the main study in Uganda, 5 semi-structured interviews were conducted among the community members. Two focus groups were also conducted amongst women’s group and CHWs in Ssisa sub county (see Additional file 1). The focus groups had 5 and 7 participants respectively. In addition, data was collected through 7 key informant interviews with health professionals, policy makers and public health researchers were carried out. Efforts were made to ensure that the interviews and focus groups were conducted in a quiet place to minimize disruptions. For the semi-structured interviews data was collected in the participant’s home or place of business. The FGDs were conducted at the THET project field office in Ssisa sub-county, and key informant interviews were conducted at participants’ offices. The interviews and focus groups were conducted in English and recorded using a digital audio recorder. A research assistant was present during the FGDs to highlight prominent themes emerging from the focus group discussions. Although incentives were not given to participants, refreshments were provided during the two FGDs.

### Data analysis

The process of data analysis followed three main steps [24]. The recorded data was first transcribed verbatim. Thematic analysis was done through manual coding of the transcribed recordings as the sample size was relatively small and manageable. A list of emerging codes was developed and collated using Excel 2013 spreadsheet. This was then followed by familiarisation and data synthesis. Identification and grouping of themes and sub-themes was done through a priori coding to align with the different levels of the socioecological framework. The coding was done by the primary author and verified by the corresponding author. As much as possible, efforts were made to ensure transcripts included emotional overtones and nuances in order to capture the full essence of the collected data was captured. These themes were then underscored by verbatim extracts.

### Validity of the study

Validity of the study was ensured through pre-testing of data collection tools, triangulation of data and transferability. A pilot study was done prior to data collection to pre-test and refine the data collection tools. To validate responses from participants, multiple methods of data collection were used, known as triangulation, to substantiate data collected [25]. To ensure transferability, the study setting was described and an audit trail [24], that is, well-documented details of the research process were provided.

The methods used in this study adhere to the Biomed Central requirement of the qualitative research review guidelines- RATS [26] in terms of appropriateness of research method, transparency of research procedure and soundness of approach.

### Ethical considerations

Prior to commencement of the study, ethical compliance was sought to protect participants from potential harm associated with the research and also protect the researchers’ interests. Ethical clearance was sought in accordance with the British Sociological Association code of ethical practice and compliance with the requirement of Nottingham Trent University School of Social Sciences Ethical Committee (Reference number: 130,315). Furthermore, ethical clearance was gained from the Makerere University School of Public Health Higher Degrees, Research and Ethics Committee and Uganda National Council for Science and Technology (UNCST) (Reference number 260315) as part of the community health workers project. A participant information sheet describing the study was provided to participants and written informed consent was obtained from each one of them before data collection. Participants personal details were not collected during data collection to ensure confidentiality and anonymity. No pictures or video recordings were taken during the FGDs, and efforts were made to ensure participants had no known history of breast cancer. Therefore, no sensitive information such as medical history was shared during the FGDs.

## Results

The findings from this study are presented under three main themes: Knowledge, Attitude, Belief and Practice (KABP); health system and policy constraints; and structural factors. These themes cut across the five levels of the socioecological framework- individual, interpersonal, organizational, community and policy. The interpersonal level has been excluded from this paper as no distinctive data were identified for this construct.

### Knowledge, attitude, belief and practice (KABP)

From an individual perspective, little or no knowledge was identified as the most significant factor that may prevent a woman from detecting breast cancer early. Almost all the participants thought that low knowledge or complete lack of it was the key barrier to early detection of breast cancer. This included low knowledge around signs and symptoms and early detection measures was the key barrier to early detection of breast cancer. Similarly, community members also identified low knowledge at primary health care (PHC) level as a key barrier as health workers, and community health workers, who are the first point of contact, focus more on communicable diseases primarily maternal health, diarrhoea and malaria. In the focus group conducted among CHWs, there was no knowledge about early detection of breast cancer. However, the majority had heard about breast cancer and cervical cancer as one of the women expressed:

*‘We don’t know how to check our breasts and I cannot know I have the breast cancer, I cannot!’* [Semi-structured #1]

In relation to attitude, apathy was highlighted as a major attitude most women exhibit, including those that may be knowledgeable about early detection of breast cancer. Although apathy is reported as an individual attitude, as in the quote below, delays in accessing health services contributes to the manifestation of this behaviour.

*‘The other thing is also attitude. Like me basically you know I have a swelling here [points to right chest area] but now I just look at myself going to the hospital and then lining up for a long time waiting. The waiting time at the hospital is also discouraging.’* [Key informant #3].

One of the key informants stated that even among health professionals who are aware and able to self-examine for breast cancer still detect it late.

*‘I have at times asked my colleagues, for example, medical people, when was the last time they examined their breasts, and you realize they have not examined their own breasts not because they don’t know but they take it [breast examination] for granted.’* [Key Informant #2].

Personal belief such as fear was also identified as a barrier to early detection of breast cancer among women. Generally, people’s attitude towards breast cancer was filled with fear. Fear was not just rooted within the immediate community but its identification by key informants suggests it was a general barrier among women. Four types of fear were identified from this study - fear of death, fear of big hospitals, fear of mastectomy and fear of the unknown.

*‘So many people even fear to go for screening because many say “why go? Because if they discover cancer I am doomed to die.” So, they have that feeling that once detected it won’t be cured.’* [key informant #4].

Participants also identified poor health seeking behaviour as another barrier. Most of the participants believed that women in Uganda only seek medical help when they feel pain. So, any noticeable abnormality in the breast may be ignored unless it is accompanied with pain. For example, one of the key informants said:

*‘Like in my experience, I realize that people go to seek health intervention when they have pain. Even when someone is just weak or they have this big swelling, if it is not painful they will leave it. When it starts paining them, that’s when they seek medical attention, and cancer of the breast by the time the lump is painful it’s already moved to another place. That is why most women don’t detect early.’* [Key Informant #2].

Other participants attributed the poor health seeking behaviour to the cultural norm in Uganda. For example, one participant stated that:

*‘We don’t have that culture of regular checks among women in Uganda. We wait for the disease to come and then rush to the hospital. So, that’s another barrier. We don’t have that health checking culture within us.’* [Key informant #5].

### Health systems and policy constraints

All the key informants agreed that health system challenges were the other big barrier hampering early detection of breast cancer not only in Wakiso district but across the country. Generally, the key informants were of the opinion that the existing health system in Uganda is not equipped to manage breast cancer, or other types of cancer for that matter, as described by one of the participants who stated that:

*‘I have always felt bad when I am telling the patient that they presented late because what did I do to help them present early? What have we done? What has the health system done to help them present early?’* [Key Informant #2].

In terms of PHC capacity, the study highlighted that the CHWs, who are the first point of contact at PHC level had no training whatsoever on breast cancer or other non-communicable diseases (NCDs) generally. Hence, they lacked knowledge on breast cancer and physical examination skills. Moving up on the health system ladder, the health facilities in Wakiso district were also reported as not equipped to address diseases such as breast cancer. Thus, individuals with abnormal signs suggesting cancer would be referred to the National referral hospital, which is the only unit for cancer that serves the whole country. One of the CHWs stated that:

*‘For me I have never got a training on breast cancer detection but I just hear that breast cancer is very dangerous and it is good for someone to go for check-ups but I have never received training on breast cancer examination.’* [FGD CHWs #7].

There was also a general perception among the community participants that health professionals at local district health facilities were inexperienced, and they could only receive quality health care from big hospitals. Key informants were also aware of this perception as one of them described it as ‘*craving for professional’s touch’*.

Another health system constraint was competing healthcare burdens. This was regarded as a major driver of public media messaging and advocacy efforts as described by the participants:

*‘I don’t think there’s been a breast cancer screening or awareness campaign done in rural parts of Uganda because there are more pressing issues. I mean there’s the WASH [Water, Sanitation and Hygiene] issue in those rural areas so why will they focus on non-communicable diseases while in the rural areas there are still communicable diseases they are trying to eradicate? I think that’s one of the major problems as to why there are not very many breast cancer campaigns done in the country generally.’* [key informant #4].

*‘Again, we are focusing more on the common diseases like malaria, cholera and typhoid. We don’t focus on breast cancer, so people are not doing these things, they don’t know about them. There’s not enough culture and there are more important diseases we are fighting with.’* [key informant #5].

This competing health burden is also reflected in the low prioritisation of NCDs generally in the teaching curricula of one of the training institutions in the country.

*‘I have to be sincere with you that the main public health component even in our curriculum is on communicable diseases, that is the focus. That’s the reality, you can’t run away from it.’* [key informant #1].

In relation to policy, a cancer policy to provide guidelines on cancer management across each spectrum of the cancer care continuum was lacking at the time of the research. The key informants confirmed the lack of cancer policy as highlighted below:

*‘There is no such policy on cancer screening or cancer prevention, there’s nothing like that.’* [key Informant #6].

### Structural factors

Participants of the FGDs and semi-structured interviews reported poverty as another barrier to early detection of breast cancer. Poverty was identified here as a structural barrier which has wider impacts on community level barriers such as transportation and individual level factors like poor health seeking behaviour. According to the FGDs participants, even if early detection services or breast cancer screening are organized, most of the women in the district would not attend because farming is their main source of livelihood and attending such breast cancer detection activities would add no ‘financial’ value to them. Therefore, this complicates health seeking behaviours and access to services. For example, one of the participants stated that:

*‘Some people will say “I am not going for breast cancer screening. They are not going to give me food so let me go to my garden and dig. Am I going to eat from there? Do they eat cancer? I don’t want to do it, let me go to my garden.” That is the mentality of our women.’* [FGD Women’s group #3].

Another barrier identified was in relation to geographical distance and how that restricts access to health services. The major challenge identified in relation to access was lack of cancer services at the PHC level. In addition to the health workers lacking training on breast cancer awareness, health facilities at the sub-county and district levels did not provide services for breast cancer detection. Although participants revealed that the UCI in Kampala usually organized weekly screening services for women, the majority may not be keen to attend because of cost of transportation. Regarding access to local services, one participant stated that:

*‘The health centre nearby the community do not offer screening services, and someone may find it hard to leave this place [Ssisa sub-county] to go to Kampala, but if they bring the services closer to the community, someone will find it easier to visit them.’*[Semi-structured #2].

The study also revealed that not only are the roads in poor condition, but travelling from the study area to the National referral hospital costs about $6 (USD). Individuals living in this community had to walk or use commercial motor bikes to the main road, where they then get on public transport to Kampala. At the time of the study, only three community organizations were identified working directly or indirectly around breast cancer, reflecting weak capacity in terms of community outreach and advocacy. Finally, participants also identified barriers related to mass media in the context of access to media platforms and knowledge construction through media reports. Generally, knowledge about breast cancer is shaped by the media. But, individuals in most of these rural areas did not have access to either newspapers or televisions, newspaper, radio or social media. Consequently, this limits the channels through which they received information on breast cancer. Community organizations suggested that stories around breast cancer are not well represented to prompt social or political actions. Furthermore, when people do read cancer stories in the media, it creates a high level of fear. Most of these stories are about the unfortunate cases who may have lost their lives to cancer or hearsay from people who did not know much about it themselves.

**Fig. 1:**
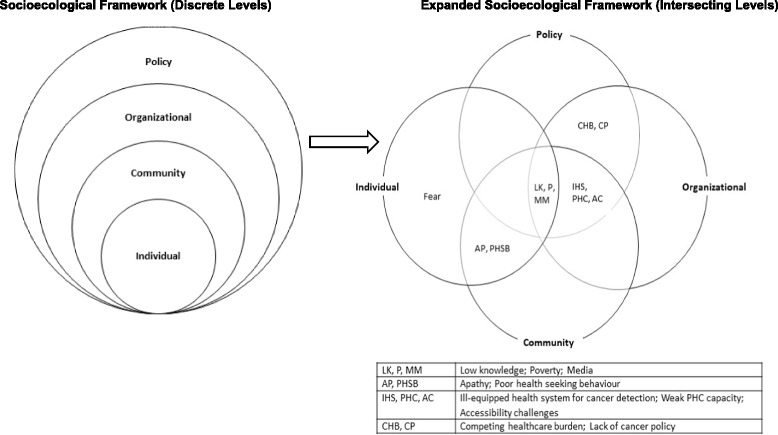
Expanded illustration of the socioecological framework showing intersections with various levels

In summary, the results from the study revealed an intricate web of interrelated factors, as highlighted in the expanded socioecological framework (Fig. 1) below. This figure shows that the different barriers within each level of the socioecological framework are not discrete. Although fear stands alone as an individual barrier, findings from the study indicated that fear is influenced and constructed by the other barriers.

## Discussion

This study examined the barriers to early detection of breast cancer in Wakiso district through a socioecological lens. Findings are consistent with existing literature that barriers to breast cancer detection in SSA are attributed to a number of complex but interacting factors including low knowledge, beliefs and inaccessibility to health facilities, and weak healthcare systems [4]. The use of a socioecological framework showed that the levels of influence are in fact non-discrete but rather intersecting. Similar to the main barriers identified in this study, Anderson et al. (2011) [27] in their summary of the 2010 BHGI consensus, also highlighted the biggest barriers of early detection in LMICs as health system and individual challenges. However, these two challenges are driven by the underlying structural factors within which both exist.

Findings from the study revealed that people were extremely fearful of breast cancer, expecting death or mastectomy after breast cancer diagnosis. This perception extends to all other types of cancer as well. Cancer generally is a dreadful word perceived as a life-threatening disease [28], both in low income and high income countries evoking myriad of negative emotions such as depression and anxiety among women [28]. Fear comes in different forms as seen in this study, but the fear of mastectomy seems to be particularly dominant among SSA women [4]. To an African woman, the breast represents more than a fashion symbol or sexual organ and is part of a woman’s identity both as a wife and mother [29]. Hence, for these women, losing the breast could result in significant loss of their identity and femininity. Fearful views such as those indicated by participants in this study can be problematic to the early detection of breast cancer. This is because such perspectives could hinder the effectiveness of cancer control as individuals are less likely to participate in cancer interventions, requiring extra motivation and efforts more than the average individual.

Previous studies have attributed the growing burden of cancer in SSA to low awareness [15]. The case of low knowledge especially intersects across the individual, organizational, community and policy levels. For instance, for the community to be aware, it has to come from the source which could either be the policy or organization. In terms of low knowledge within the organizational level, the study revealed that CHWs, who are crucial to PHC delivery, lack knowledge about early breast cancer detection. Similar to findings from this study, Morhason-Bello, et al. [15] indicated that health workers at the primary care level are only equipped to provide awareness or in some cases first point of care on basic communicable diseases. For example, a study conducted in Nigeria revealed that even professional health workers such as nurses at community level had low knowledge regarding general breast awareness [30]. Low knowledge also operates within the policy realm as a driver of political will and commitment towards addressing breast cancer detection. As indicated in this study, Uganda had no cancer policy and this could reflect low priority for the disease generally.

In addition to low knowledge, the attitude and practice of women towards breast cancer detection is very relevant. However, these are not only as a result of individual behaviour but can build up as a norm across the community over time. Besides, structural factors such as distance, waiting times and cost of transportation may influence the attitude of a woman towards going for breast cancer screening at the National referral hospital. In relation to practice, the major barrier identified from this study was poor health seeking behaviour among women. Poor health seeking in breast cancer detection behaviour goes beyond an individual barrier and can be regarded as a community factor which has been identified by other studies in other countries such as in United Arab Emirates [31]. Studies in Uganda have also described that generally health seeking behaviour among Ugandans are poor and deeply rooted in community norms and practices [32] almost regarded as a culture. The fact that breast cancer is a disease that is not necessarily associated with pain at the onset makes it difficult to detect early. As alluded in the study, women will most likely seek medical intervention when they feel pain. Effective awareness and context specific health promotion messages would be relevant in this instance to provide adequate information on breast cancer symptoms and how they present, particularly the need to have any abnormality checked out early at primary healthcare level.

It is well known that the challenge of poverty in Uganda and other SSA countries results in low prioritisation of breast cancer and other diseases [33]. The CHWs revealed that those women who were engaged in activities to raise income for themselves and their families, especially farming, were less likely to be involved in any form of early detection strategies, including BSE. Previous studies focusing on low resource countries have also recognised the impacts of poverty and livelihoods as burdens which prevent women from attending to their personal health [34]. Livelihoods prevent women from having the time for attending to their personal health as identified by Muthoni and Miller [35] in a qualitative study carried out to assess the knowledge and attitudes of Kenyan women regarding breast cancer detection. Therefore, this complicates their motivation to attend screening services or seek medical attention early.

More often than not, PHC in many LMICs including Uganda is still focused on communicable diseases [30]. An effective referral pathway is not in place in most countries. Therefore, individuals rely on higher health services such as national hospitals as they are aware that the PHC facilities lack capacity to provide information on management of breast cancer [15]. This inadvertently strains the tertiary care facilities [36] thus reducing their performance. Given these findings, there is need for a more rigorous study on how PHC capacity can be strengthened to deliver early detection services in low resource settings particularly focusing on CHWs as they represent the link between community members and the medical health system. One strategy for this could be the training and education of PHC workers. This is an essential component of the national cancer control programmes guidelines put in place by the WHO to promote national policies and strategies to effectively address the cancer burden [37].

This study also revealed that breast cancer and other NCDs are generally not prioritised in SSA countries such as Uganda. This is mainly as a result of the high burden of communicable diseases in SSA, giving rise to what has been termed the double burden of diseases [38]. It was evident from this study that the country’s health system lacks the capacity to address the growing burden of cancer and other NCDs. This is understandable, given that there is still a huge burden of communicable diseases such as malaria and HIV/AIDs in the country and other parts of SSA [15]. Ill-equipped health systems at the community level reflects inadequate community advocacy and grassroots efforts to drive prioritisation of breast cancer and other NCDs. This case is not only unique to the study area but cuts across other SSA countries where community centred cancer advocacy strategies are still considered inadequate [15].

The lack of a clear cancer policy in Uganda also complicates this problem of early detection as there is no guidance on how the health system should address cancer control. The existing health policy documents, the Uganda National Health Sector Strategic and Investment Plan III 2010/11–2014/15 [39] and National Health Policy II 2010–2020 [40] do not refer to breast cancer. However, the National Health Sector Strategic and Investment Plan III 2010/11–2014/15 acknowledged a survey on the magnitude on NCDs in Uganda had not been conducted because of funding challenges. This lack of guidance inadvertently contributes to the late detection of breast cancer as there are no policy requirements for early detection at primary health facilities. Thus, this creates significant geographical barriers in terms of access to services and increase the strain on the National referral hospital. Typically, resource allocation for healthcare management tends to be driven by political goals [5] which are reflected in the nature of health policies produced. This indicates that a political commitment to addressing the growing burden of cancer is crucial to drive organizational change within the health system, which will consequently feed into the other levels even to the point of knowledge awareness to the individual.

### Limitations of the study

This study involved only one sub-county, therefore the results may not necessarily be generalizable. Despite being carried out in one sub-county, key informants were recruited from other areas such as the National referral hospital and training institutions. While the study may not be generalized as an indication of the breast cancer situation in the whole of the country, it is a good representation of what may most likely be the case in most parts of Uganda and similar contexts.

## Conclusion

Early detection of breast cancer is a key aspect of management of the disease. However, women in Uganda usually detect breast cancer late as a result of complex but interacting barriers. These barriers associated with KABP, health system and policy constraints and structural challenges intersects with multiple levels of the socioecological framework. The synergistic interactions of these barriers make early detection of breast cancer difficult in Uganda.

Clearly, investments to improve the health system capacity in Uganda is required with particular focus on strengthening PHC capacity to promote and ensure universal coverage in terms of breast awareness In addition, these findings provide opportunities for policy and practical interventions in breast cancer management, particularly through coordinated efforts and investment in multi-level interventions. In view of these findings, health promotion interventions seeking to improve practices regarding early detection of breast cancer should take a multi-level approach with consideration of the prevailing socio-cultural and political contexts within which the challenge of breast cancer is constructed.

## Additional file

Additional file 1: FGD Guide. The focus group discussion guide used to collect data from both women group and CHWs. (DOCX 16 kb)

## Abbreviations

BHGI: The Breast Health Global Initiative
BSE: Breast self-examination
CBE: Clinical breast examination
CHWs: Community health workers
FGDs: Focus group discussions
KABP: Knowledge, attitudes, beliefs and practices
NCDs: Non-communicable diseases
PHC: Primary health care
SSA: Sub-Saharan Africa
THET: Tropical Health Education Trust
UNCST: Uganda National Council for Science and Technology
WASH: Water, sanitation and hygiene
WHO: World Health Organization
