# Supplementary material for: Perceived barriers to early detection of breast cancer in Wakiso District, Uganda using a socioecological approach
Source: Global Health. 2018 Jan 23;14:9. doi: 10.1186/s12992-018-0326-0 (PMC5781279; doi:10.1186/s12992-018-0326-0)
Supplement: Additional file 1: — FGD Guide. The focus group discussion guide used to collect data from both women group and CHWs. (DOCX 16 kb) [file 12992_2018_326_MOESM1_ESM.docx]

**FOCUS GROUP 1 - WOMEN**

**Introduction**

- General introductions
- About the project

**Awareness of breast cancer and early detection of breast cancer**

- What do you know about breast cancer (what is it? Who gets it? What are the risk factors? Signs/symptoms? Age band? Conceptions/misconceptions about the disease?)
- What do you know about early detection of breast cancer? (What is it? Why is it done? How is it done? At what age?)
- What breast cancer detection services are available? How accessible are these services?
- How do the communities engage with these services in terms of uptake?

**Barriers questions**

- From your perspectives, what factors might prevent a woman from detecting breast cancer early? (Cultural beliefs, lack of knowledge about breast cancer/breast cancer detection etc)?
- Can you please describe the kind of support available (if any) to encourage early detection of breast cancer? (Family support, transport, etc).
- Are there any organisations that work around breast cancer awareness in your community? (Who are they? How do they create awareness? - Media, posters, campaigns etc).

**Suggestions to improve early detection**

- What strategies/activities/interventions would you suggest be put in place to improve early detection?

**FOCUS GROUP 2 - COMMUNITY HEALTH WORKERS (WOMEN)**

**Introduction**

- General introductions
- About the project
- Please tell me about yourself and your role as a community health worker.
- What kind of training have you received in this role? Particular training regarding non-communicable diseases? Any on breast awareness or breast cancer awareness?
- How are health messages passed across to the community (eg training, leaflets, community gatherings etc)?

**Breast cancer/breast cancer detection**

- What do you know about breast cancer (what is it? Who gets it? What are the risk factors? Signs/symptoms? Age band? Conceptions/misconceptions about the disease?)
- From your perspectives, how aware would you say women in the community are about breast cancer? (reasons- discussion)
- What do you know about early detection of breast cancer? (What is it? Why is it done? How is it done? At what age?)
- What breast cancer detection services are available? How accessible are these services?
- How do the communities engage with these services in terms of uptake?
- Can you discuss existing health promotion practices (if any) in relation to early detection of breast cancer (Who is driving these activities)?

**Barriers questions**

- From your perspectives, what factors might prevent a woman from detecting breast cancer early? (Cultural beliefs, lack of knowledge about breast cancer/breast cancer detection etc)?
- Can you please describe the kind of support available (if any) to encourage early detection of breast cancer? (Family support, transport, etc).
- Are there any organisations that work around breast cancer awareness in your community? (Who are they? How do they create awareness? - Media, posters, campaigns etc).
- Do you know of any policies related to breast cancer?

**Suggestions to improve early detection**

- What strategies/activities/interventions would you suggest be put in place to improve early detection?
